# Is air pollution detrimental to regional innovation? An empirical heterogeneity test based on Chinese cities

**DOI:** 10.3389/fpubh.2022.981306

**Published:** 2022-11-21

**Authors:** Zhilin Liao, Mingxing Hu, Lei Gao, Baodong Cheng, Chenlu Tao, Rizwan Akhtar

**Affiliations:** ^1^School of Economics and Management, Beijing Forestry University, Beijing, China; ^2^School of Economics and Management, Yanshan University, Qinhuangdao, China; ^3^School of Economics and Management, North China Electric Power University, Beijing, China; ^4^Department of Economics, Karakoram International University, Gilgit, Pakistan

**Keywords:** air pollution, regional innovation, regional heterogeneity, occupational health, crowding out effect

## Abstract

Nowadays, innovation seems to be the inevitable choice to achieve stable economic growth. However, the negative impact of air pollution on health and economy makes air pollution an important factor in regional innovation, which deserves our discussion. The overall regional innovation level from 2014 to 2019 has an upward trend, while the overall air pollution has a downward trend during the period, which provides foundation for our research. Based on the data of 285 prefecture-level cities in China from 2014 to 2019, this paper uses the fixed effect and mediation model to verify the impact and mechanism of air pollution on regional innovation. The results show that the increase in air pollution, measured by the air quality index, significantly inhibits regional innovation. Air pollution has significant funds crowding-out effect and human capital loss effect, thereby decreasing the regional innovation level, which means innovation funds and researchers play a conductive role between air pollution and regional innovation. In heterogeneity analysis, it is found that the detrimental effect of air pollution on regional innovation is significant in eastern and central China, in large- and medium-sized cities, and in cities with poor or general air quality. It indicates that developed and large-scale regions should pay more attention to air pollution control. For polluted regions, more emphasis and endeavors are needed to address air pollution problems. Besides, the inhibitory effect is more severe on incremental innovation rather than on radical innovation, which deserves the attention of enterprises engaged in incremental innovation. Therefore, we propose that targeted environmental policies and effective measures should be developed to improve air quality in the long run. Moreover, policymakers could provide strong support for innovation grants, talent subsidies, and rewards and encourage clean technological innovation through short-term trade-offs between heavily polluting and low polluting enterprises.

## Introduction

With economic globalization and extensive economic growth in recent years, the global ecological environment is deteriorating day by day, which poses a challenge to the sustainable development of the world's economy and environment (1). Due to the extensive economic growth, the consumption of resources and energy has increased (2), the level of biodiversity has decreased, and the contradiction between human habitat activity and ecological environment has become increasingly prominent (3). As a result of extensive economic growth (4), air pollution has become a global city disease in contemporary society threatening both human lives and economic development (5, 6). According to the World Health Organization (WHO), air pollution is one of the main environmental hazards to human health. The WHO reported that nine out of ten individuals inhale polluted air and that seven million people die every year because of these dangers. The United Nations Environment Program reported that air pollution in developing nations had increased the financial burden by approximately 5% of GDP (6), which poses a challenge to China's sustainable development (7, 8). In 2018, China's environmental performance ranked 120th in 180 countries around the world, and its air quality ranked 177th (fourth from bottom). According to the 2018 statistical bulletin of the Ecology and Environment of China, the air quality in 217 of 338 cities at the prefecture level or above fails to reach the standard, accounting for 64.2% of all cities. Due to the serious impact of air pollution, it has gradually become one of the key regional factors highly valued by relevant departments and local governments (9–11). To confront the severity of air pollution, the Chinese government also has formulated a series of environmental regulation policies (12, 13). For example, Five Development Concept was proposed, including innovation and green. In 2013, China promulgated the China Air Pollution Prevention and Control Action Plan (14), gradually shifting its focus from rapid economic growth to efficiency and technology-related driving forces. In March 2017, the Chinese government proposed to make skies blue again in the Report on the Work of the Government (15).

Due to the severe impact of air pollution on human health and economy, air pollution becomes one of the non-negligible factors to regional innovation. In recent years, innovation has been regarded as one of the key factors to speed up the replacement of the old drivers of growth (16) and achieve sustainable development (17, 18). Regional innovation refers to the technological innovation in a certain region (19), which mainly indicates a prefecture-level city in this paper. Regional innovation generally has two types, namely, radical innovation and incremental innovation (20). Radical innovation is the most important type of innovation, which represents novelty, creativity, and practicality. Table 1 displays the specific types and applications of regional innovation. As the exploration of unknown paths, radical innovation and incremental innovation generally have the characteristics of long cycle, great uncertainty, high failure rate, but huge potential benefits (21). It is widely known that innovation is affected and restricted by many factors. At the national level, innovation needs high-quality talent supply (22) and huge financial support (23) combined with active government guidance (24). At the enterprise level, enterprises, as the main subjects of innovation, are faced with high sunk costs, lack of innovation incentives, and insufficient information and technical support (25, 26). For patent inventors, innovation is seriously restricted by individual factors and environmental factors to some extent. Individual factors include personal characteristics, personal intellectual capital, and so on, while environmental factors mainly refer to organizational culture and team behavior (27). It cannot be ignored that, in the context of China, severe environmental problems, especially poor air quality, may have impacts on the regional innovation level (28, 29); however, the research is insufficient (30–32). Most of the related research is concern of the role of technological innovation in air pollution based on the Porter hypothesis (PH) but ignores the impact of air pollution on technological innovation. First, health issues are constantly rising due to air pollution especially in developing countries (33), which leads to the loss of labor supply and the dampening of work efficiency of regional innovation (34). Moreover, to avoid further deterioration of air pollution, the Chinese government also has formulated a series of environmental regulation policies, which increase the cost of enterprises (35). Therefore, studying the effect of air quality on innovation and its impact mechanism will help to comprehensively understand the negative effects of air pollution and remove obstacles to regional innovation.

**Table 1:** Types and applications of regional innovation.

**Type**	**Definition**	**Applications**	**Application city**
Radical innovation	Innovation with fundamental and significant technological changes. It is often accompanied by a series of progressive product innovation and process innovation	A control method and device for switch-type electro-pneumatic valve of electric multiple units Power integration system arranged under the vehicle of High-speed Diesel Multiple Unit A signaling configuration system and method for measuring reference signals	Tangshan Dalian Shenzhen
Incremental innovation	Local or benign innovation for process or product. The most extensive form of innovation, because the risk-taking cost seems low and the benefits are considerable	Construction technology of directly burying prefabricated block of indoor box shell Artificial intelligence cloud diagnosis of cervical cancer An outdoor live working robot manipulator for automatic tool exchange	Shenzhen Wuhan Nanjing

The motivation of the study was to clarify the relationship between air pollution and regional innovation, design a better way to stimulate innovation, meanwhile identify the transmission mechanism, and find a solution to control air pollution and improve innovation level. Many studies found the positive effects of technological innovation on the reduction in pollutant emissions (36, 37). However, does environmental pollution influence technological innovation? One potential problem in the literature is a lack of research on the impact of air pollution on innovation. Most studies focus on the one-way effect of technological innovation on environmental pollution from the perspective of industry, enterprise, or individual emotion. There are few studies on the impact of environmental pollution on technological innovation at the regional level. Clarifying the specific impact of air pollution on regional innovation will help to test the efficiency of government policies and provide suggestions for further improving the regional innovation level and economic development quality. The goal of this research was to clarify the transmission mechanism and provide possible paths to block or reduce the impact of air pollution on innovation for regions or countries unable to solve air quality problems in the short run. To this end, based on the number of patents granted and air quality index (AQI) data of prefecture-level cities from 2014 to 2019, this paper uses the fixed-effect model, establishes the influencing model of air pollution to unravel the relationship between them, and analyzes the specific impact of air pollution on regional innovation by considering regional heterogeneity.

The possible contributions of this paper are as follows: First, previous studies generally focus on the relationship at the provincial level, whereas some studies refine to the heavily polluted cities at the prefecture level. This paper refines the research data of air pollution on regional innovation and expands the research area to the whole country at the prefecture level, taking the data of 285 prefecture-level cities as an example. To some extent, this study serves as a basis and new mentality for further research on the subject due to the few studies in this area. Second, this paper discusses the impact of air pollution on regional innovation from the level of prefecture-level cities, constructs the theoretical framework of air pollution and regional innovation, and makes theoretical research and empirical test on their impact mechanism. The clarification of transmission mechanism could provide possible paths to block or reduce the impact of air pollution on innovation for regions or countries unable to solve air quality problems in the short run. This paper examines the human capital loss effect through the number of researchers not the whole labor, which makes the effect more accurate. Third, this paper discusses the effects of air pollution on regional innovation according to the heterogeneity of development level and city size, which provides basis for the formulation of innovation policies of different cities.

## Evolutionary characteristics of air pollution and regional innovation

**Figure 1:**
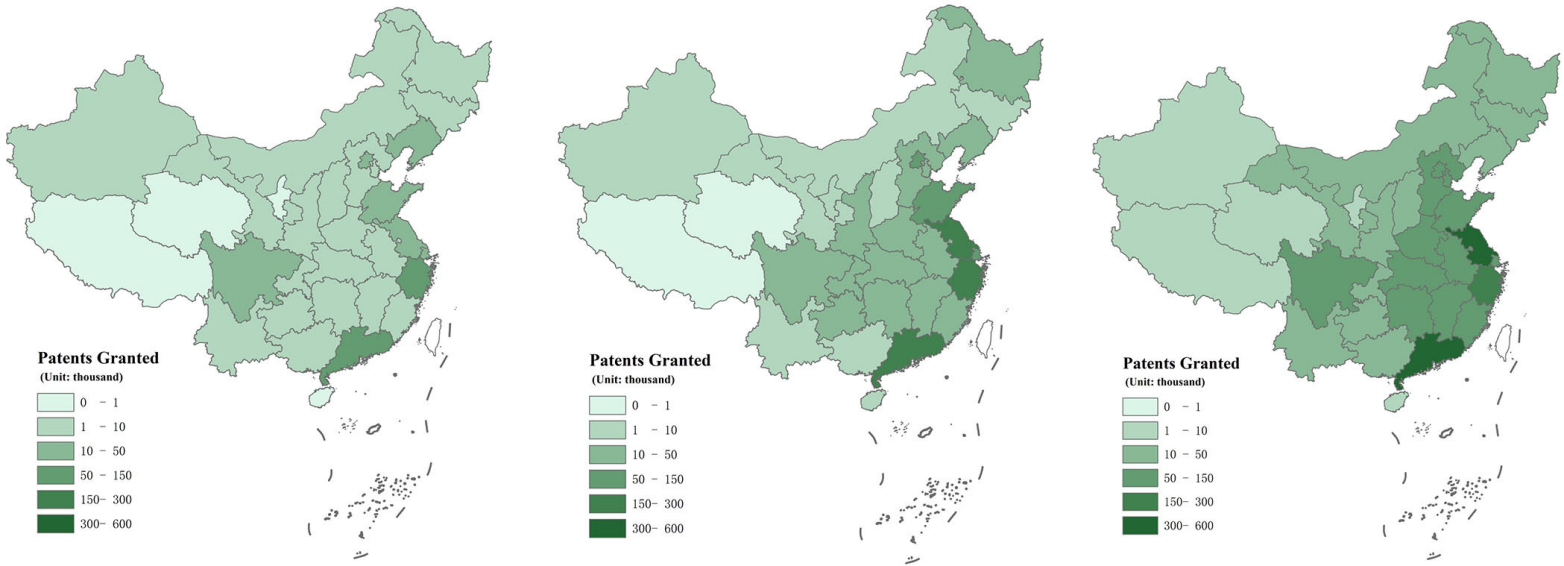
Maps of China's regional innovation in 2008, 2014, and 2019.

**Figure 2:**
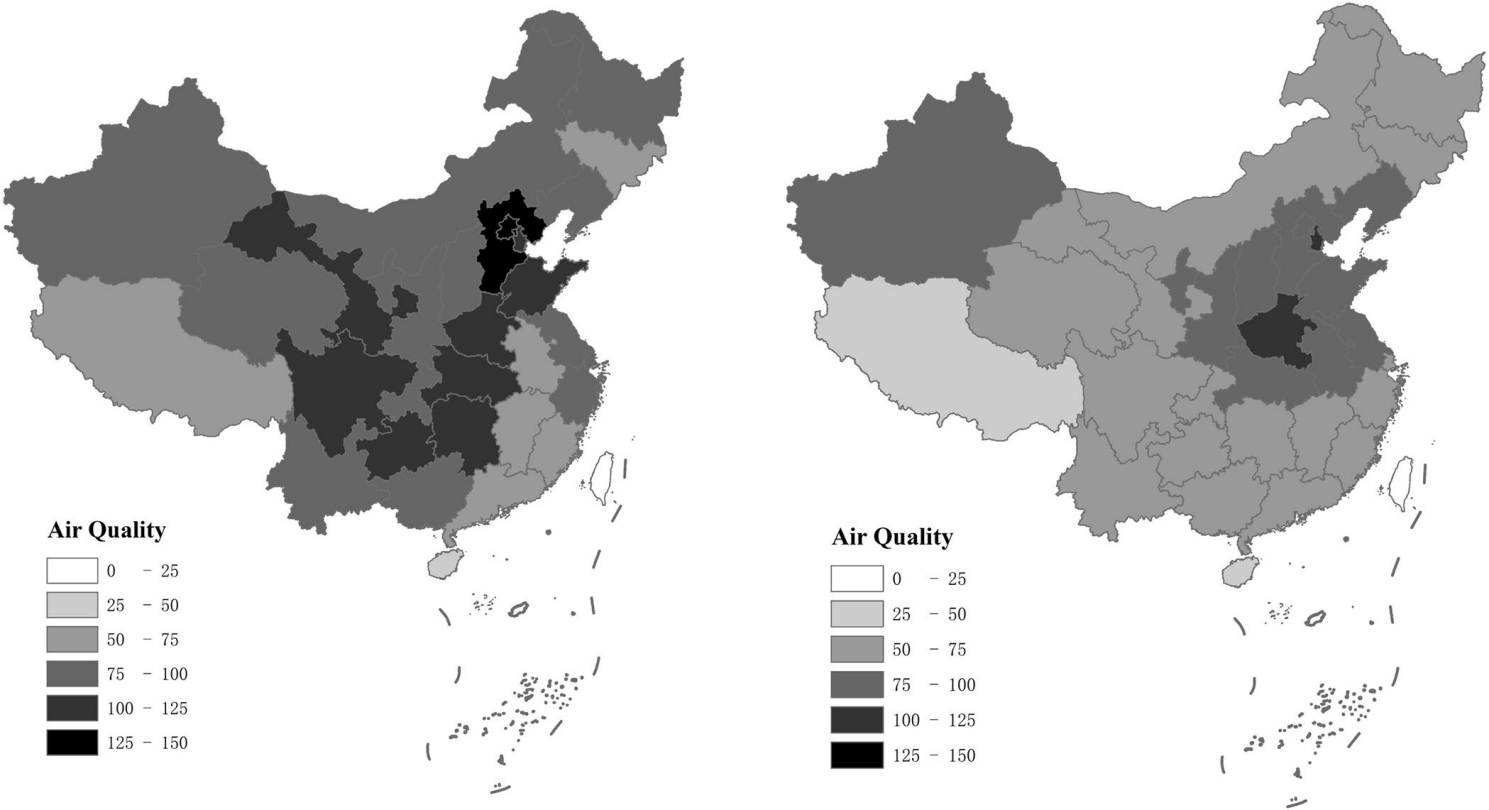
Maps of China's air pollution in 2014 and 2019.

Maps are drawn to analyze the spatial characteristics of air pollution and regional innovation. Figure 1 shows the maps of China's regional innovation in 2008, 2014, and 2019, respectively. As can be seen in the figure, in 2008, only a few provinces had more than 50,000 patents, namely, Zhejiang and Guangdong. In 2014, the number of provinces with more than 50,000 patents increased to 6, including Jiangsu, Zhejiang, Guangdong, Beijing, Shandong, and Shanghai. In 2019, 15 provinces had more than 50,000 patents, accounting for almost half of China. Among them, the province with the highest number of patents is Guangdong, with the total number of 527,390. The total number of patents is increasing year by year, which indicates the overall regional innovation level among 285 prefecture-level cities has an upward trend from 2008 to 2019, and the regional innovation in eastern China grows rapidly. The average increase in patents granted exceeds 150%. Benefitting from innovation incentive policies of central and local governments, the number of patents is growing rapidly in these years. Besides, Figure 2 shows the maps of China's air pollution in 2014 and 2019, respectively. Because the index AQI was proposed at the end of 2013, this paper only selected two years to reflect the evolutionary characteristics of air pollution. The reduction trend of air pollution can be seen clearly from the figure. In 2014, there were 10 provinces with serious air pollution (air quality index greater than 100), namely, Hebei, Beijing, Tianjin, Gansu, Shandong, Henan, Guizhou, Sichuan, Hubei, and Hunan. However, in 2019, only two provinces suffered severe air pollution (air quality index greater than 100), namely, Henan and Tianjin. The overall AQI among 285 prefecture-level cities has a downward trend from 2014 to 2019, with an average decrease of 16%. It means the average air pollution has decreased by 16% over 6 years. In 2013, China promulgated the China Air Pollution Prevention and Control Action Plan (14), gradually shifting its focus from rapid economic growth to efficiency and technology-related driving forces. In March 2017, the Chinese government proposed to make skies blue again in the Report on the Work of the Government. Benefitting from these air pollution control policies, China's air quality has improved in recent years, but some areas are still plagued by air pollution. We observed that the areas with high innovation levels are mostly the areas with less serious air pollution. Intuitively, the possible reason is that air pollution has an impact on innovation. Next, we will verify the relationship between air pollution and innovation from both theoretical and empirical aspects.

## Literature review and research hypothesis

### Literature review

#### Empirical studies on innovation

Numerous scholars have studied the factors affecting innovation and further focused on regional innovation. The input of research and development (R&D) resources, the degree of competition and coordination among innovation subjects, and the support of government and market remain the focus of studies. First, the input of R&D resources has a profound impact on the output and performance of regional innovation. Xin et al. (38) explored the effect of R&D intensity on technological innovation and the moderating role of free cash flow using negative binormal regression models. Xu et al. (39) found that government subsidies play a vital role in enhancing firms' investment in research and development (R&D) and R&D investment has a positive impact on innovation performance. Second, the degree of competition and coordination among innovation subjects may drive regional innovation to a high level. Collaborative innovation and spatial correlation are expected to contribute to regional innovation performance. Li and Xing (40) found that the innovation collaborative agglomeration and the regional innovation capacity present a typical inverted U-shaped relationship in the stage of knowledge innovation, while there is a U-shaped relationship between the innovation collaborative agglomeration and the regional innovation capacity in the outcome transformation stage (41). Third, regional innovation requires the support and encouragement of the government. Overall, government subsidies can improve regional innovation quality (42), which is mainly realized by increasing the input of innovation resources from local direct innovation subjects, attracting the inflow of innovation resources from neighboring areas, and increasing the innovation support from local indirect innovation subjects (43). Porter and Linde (44) proposed that appropriate environmental regulation could prompt profit-maximizing enterprises to increase technological innovation to reduce their environmental costs and then achieve the improvement in economic performance.

#### Air pollution and economic growth

Academic circles have triggered a profound discussion on the trade-off between economic development and air pollution. The environmental Kuznets curve (EKC) theory exposes a connection between economic growth and environmental degradation (45, 46). The EKC hypothesis defines the relationship between economic development and environmental deprivation, and this association influences once a specific income limit is accomplished (47). Moomaw and Unruh (46) studied the affiliation of CO_2_ emissions with GDP in 16 countries over 1950–1993 and found the N-shaped EKC with addition to structural transition. Fodha and Zaghdoud (7) studied the relationship between air quality and economic growth in Tunisia, a North African country, which is suitable to be described by monotonic growth. Yang et al. (3) examined that remittance and energy use increases CO_2_ emissions; however, globalization reduces CO_2_ emissions. Besides, remittance inflows significantly enhanced the environmental degradation in 5th to 70th quantiles for the global panel of 93 countries and it turns to becomes negative at 80th to 95th quantiles, which also support the validity of an inverted U-shaped EKC hypothesis (48). Awan et al. (49) found that globalization and financial development have adverse and significant effects on environmental degradation and affirm the legitimacy of the EKC hypothesis for these countries. However, there is an inverted U-shaped relationship between economic growth and CO_2_ emissions in China (50). Therefore, the relationship between air pollution and economic development cannot be described in a single dominant form (51).

Specifically, researchers are mainly concerned about the one-way effect of economy on air pollution. The pollution haven hypothesis argues that globalization and the removal of trade barriers lead to the relocation of environmental harmful production staging from high-income countries with stringent environmental regulation to developing countries with less stringent environmental regulation and therefore aggravate the air pollution in developing countries (52). Kamal et al. (53) investigated that the marginal impact of fiscal policy on carbon emissions is 0.0017% in the long run, which indicated that fiscal policy significantly increases environmental pollution. Moreover, Yang et al. (54) determined the dynamic linkages between globalization, financial development, and carbon emissions in Asia Pacific Economic Cooperation (APEC) countries, with the result that globalization and financial development significantly reduce carbon emissions by 0.033 and 0.0021%, respectively, but economic growth and energy intensity increase them; more specifically, a 1% increase in economic growth will lead to an increase in the carbon emissions by 0.841% (54), which supports the pollution haven hypothesis (55). Besides, autocracy, political globalization (56), economic growth, financial development (55), energy consumption, and gross fixed capital formation significantly enhance environmental degradation, while democracy, economic, social, and overall globalization significantly reduce it (56).

Some scholars also pay attention to the one-way effect of air pollution on economic growth. Relevant studies mostly focus on the generation of air pollution, the impact of air pollution on human health, and the role of pollution on economic growth. First, air pollution will greatly affect residents' health, with the estimation for all patients 1.023 and a short period <3 days (57). Air pollution has a significant inhibitory effect on residents' self-rated health and life satisfaction (58). Apergis et al. (59) discovered that a 1% increase in carbon emissions increased health expenditure by 2.5% in four global income groups of 178 countries between 1995 and 2017 and 2.9, 1.2, 2.3, and 2.6% in the low-, low-middle-, upper-middle-, and high-income groups, respectively. Azam and Awan (6) indicated that air pollution has a long-run and significantly positive relationship with healthcare costs, a 1% reduction in air pollution will result in a 1.138% reduction in healthcare expenditures, and developing countries are more likely to be affected by the climate change and thus have to face the problem of increasing healthcare expenditures (6). Second, air pollution may lead to the loss of labor force (60), especially the loss of high-skilled labor (61). When the labor force faces the perceived risk of air pollution, it is willing to choose the migration way and invest in health capital, so as to give play to the substitution effect of other health investment modes and the avoidance effect of health damage, and thus effectively improve its own health status (60, 61). Third, air pollution may cause a distortion in Chinese labor wage, especially neighboring air pollution, which is the spatial spillover effects of pollution, with a 1% increase in air pollution leading to a 0.0842% and 0.1038% increase in wage distortions and marginal product labor, respectively (62). Moreover, air pollution has gradually inhibited the improvement in comprehensive urbanization, population urbanization, economic urbanization, and living conditions urbanization (63).

#### Air pollution and innovation

The impact of air pollution on regional innovation attracts some academic attention. Most studies focus on the impact of air pollution on innovation from the aspect of enterprise. In general, air pollution has both “inhibition effect” and “incentive effect” on technological innovation (64–66). The former means that air pollution will trigger strict environmental regulations, increase business costs, and inhibit technological innovation (67). The latter refers to the fact that air pollution will stimulate industrial transformation and innovation, thereby promoting technological innovation (68). Lin et al. (8) investigated the influencing mechanism and effects of air pollution on technological innovation from the perspective of innovation value chain, who found that air pollution has a significant crowding-out effect on innovative funds with a coefficient of −0.0758, thereby decreasing the innovation output by 0.0973%. Air pollution collaborative governance increases the number of green utility model patents, with a 1% increase in air pollution collaborative governance leading to a 0.0497% increase in green utility model patents, but has no obvious impact on green invention patents (69). For regional innovation, air pollution may significantly reduce the level of regional innovation and inhibit the output of regional innovation. It has a more and more severe negative impact on innovation efficiency at the urban level, specifically, a 1% increase in PM_2.5_ concentration will result in a 0.042% reduction in patents (19), and the channels through which air pollution affects urban innovation are mainly attributed to production efficiency, willingness to consume, and entrepreneurial activity (14). Besides, affected by the “resource crowding-out effect,” the negative impact of air pollution on urban innovation capability in key environmental protection cities is far greater than that in non-key cities (70). Among them, PM_2.5_ inhibits regional innovation significantly with the coefficients of −0.141 for internal effect and −0.083 for external effect, and this result still exists after using the air mobility index as an instrument variable to alleviate endogenous problems (71).

Our literature reviews reveal the following deficiencies in existing studies. First, there are only a few studies investigating the effect of air pollution on technological innovation, or their bi-directional relationship at the regional level, mainly from the provincial level, and the data are relatively macro. But the prefecture data further consider the heterogeneity of different cities in the same province, and the results are more realistic and representative. However, as heavily polluted cities tend to be the main focus of prefecture-level research, there is a lack of more universal research, which leaves space for this paper. Second, previous research exploring the influencing mechanism of air pollution on technological innovation mainly explores the crowding-out effect on innovative funds, while few studies exploring the crowding-out effect on labor, if any, mainly focus on all labor, not the number of researchers. Third, previous studies seldom consider the city size heterogeneity neither the situation whether the air pollution affects regional innovation differently due to the air quality conditions. As the essential environment for human survival, it deserves our research whether and how air quality affects regional innovation.

### Theoretical framework and research hypothesis

#### Theoretical foundation

The theoretical foundation of this paper is crowding-out theory and labor force heterogeneity theory.

Crowding is regarded as a negative phenomenon relating to social density (72). Crowding-out effect means that in a relatively flat market, due to the new increase in supply and demand, some funds are squeezed out of the original expenditure and flow into new commodities (73). Although environmental policies may be crucial to avoid the socio-economic cost of environmental disasters, economic restriction is also concerned not to threaten competitiveness of the business sector. Innovation has long been understood to be an essential driver of such competitiveness (74, 75). For a long time, environment pollution and restriction, especially environmental regulation, have been particularly suspect to being a source of crowding-out effects.

The theory of labor force heterogeneity provides a new perspective for the green development of enterprise human capital (76, 77). Air is a kind of public goods (78); without considering the externality, the response of enterprises to the deterioration of air quality and the treatment of their own excessive exhaust emissions are only part of their investment allocation and strategic choice. In essence, an enterprise is mainly composed of a series of human capital (79). The guarantee of employees' physical and mental health constitutes an important part of this incomplete contract (80). Although air pollution will bring a series of financial and human capital pressures to enterprises (81), enterprises constrained by budget cannot cope with the negative consequences of external air quality deterioration in all aspects at the same time. Based on the theory of labor force heterogeneity, different from other relatively homogeneous human capital, skilled employees are no longer just labor elements allocated by enterprise managers, but have higher independent choice and labor market negotiation power under the conditions of modern technology (82). Therefore, they are likely to take the lead in “escaping from smog” when facing the pressure of air pollution that endangers their body and mind (83).

#### Theoretical framework

**Figure 3:**
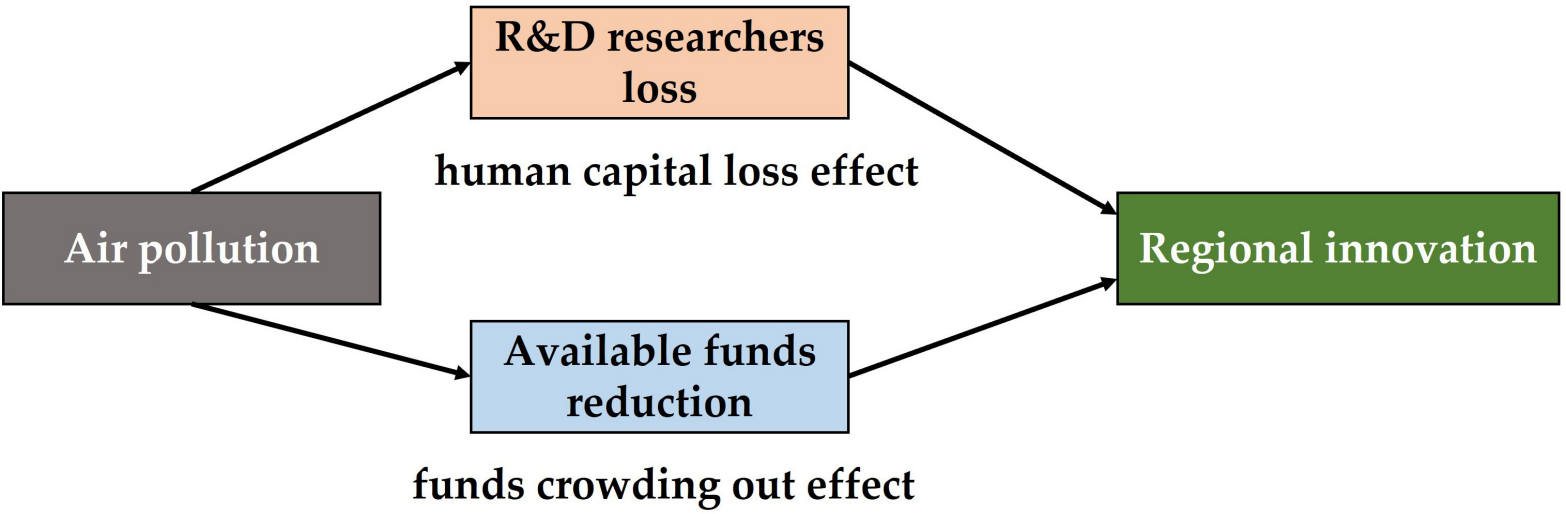
Transmission paths of air pollution to regional innovation.

Based on the above theories, this paper puts forward two transmission paths, namely, “human capital loss effect” and “funds crowding-out effect.” Figure 3 displays the transmission paths of air pollution to regional innovation. As for the “human capital loss effect,” air pollution mainly affects human resources through “employee health” and “employee mobility,” which makes human capital unable to play an effective role in innovation, and then exerts a negative impact on urban innovation. The “funds crowding-out effect” is mainly manifested in the increase in internal pollution control cost and external pollution prevention cost. The additional investment of manpower, financial resources, and technology is a kind of resource squeeze for innovation development, which is not conducive to the improvement in innovation capability.

First, air pollution may lead to dampening the enthusiasm to innovation of labor force and the loss of labor force (84). Air pollution affects the health of residents, and the incidence rate of respiratory diseases of residents in the haze weather has greatly increased. With a long-term exposure to severe air pollution environment, the life span of researchers will be reduced (85), and the risk of disease and the probability of early death will be increased (86). The innovative talent is the core element of innovation activities. Considering the harm of air pollution to the health, some people, especially scarce high-quality innovative researchers, will leave heavily polluted places (87). They have strong incentives to migrate or leave due to factors threatening their health, resulting in the loss of innovative researchers, and then inhibiting the generation of innovation. Besides, air pollution may influence the efficiency of work and productivity of individuals, which hinders researchers from innovating (83).

Second, the severity of regional air pollution is likely to make the local government increase the requirements and punishment mechanism for environmental governance (88), which reduces the available funds of enterprises and then forces enterprises to reduce investment in R&D and innovation. Besides, air pollution may also damage the health of enterprise employees; thus, they may require higher wage returns (89), which also increases the operating costs and reduces the available funds. The reduction in the available funds may eventually lead to enterprises unwilling to enter or decide to relocate, for the purpose of minimizing the impact of environmental costs (90). The weakening of the innovation capability of enterprises and the loss of enterprises finally tend to lead to the decline of the overall regional innovation capability.

Therefore, this paper develops the hypothesis (H) as follows:

H1 Air pollution has a negative impact on regional innovation.
H2 Air pollution negatively affects regional innovation through the human capital loss effect.
H3 Air pollution negatively affects regional innovation through the funds crowding-out effect.

The following study will devote to testing the hypothesis.

## Methodology

### Data

This paper selected AQI and the number of patents granted data of 285 prefecture-level cities in China from 2014 to 2019, which obtains 1,562 observations. In terms of AQI, referring to the research of Li et al. (91), the daily AQI of prefecture-level cities was obtained from the National Air Quality Daily (https://datacenter.mee.gov.cn), and the annual average was carried out to obtain the annual AQI. The number of patents granted is from China City Database, which is used to measure the innovation level of 285 prefecture-level cities.

The relevant data of prefecture-level cities are from China City Database, and other data including GDP and the number of industrial enterprises above designated size are from the National Bureau of Statistics, while the data of local government scientific research expenditure and the amount of foreign capital used are from China City Statistical Yearbook.

### Definition of main variables

Table 2 displays the characteristics of all variables, and the main variables are defined in the following.

**Table 2:** Characteristics of all variables.

**Symbols**	**Variables**	**Unit**	**Definition**
inno	Regional innovation	Piece	The technological innovation in a certain region, which indicates a prefecture-level city in this paper
Patent	Patents granted	Piece	The proxy variable of regional innovation, which is the number of patents granted in the prefecture-level city. There are three types of patents: patent for invention, patent for utility model, and industrial design
AQI	Air quality index	Numerical	A dimensionless relative value that comprehensively represents the degree of air pollution, calculated from six air pollutants with unified evaluation standards, including PM_2.5_, PM_10_, SO_2_, CO, NO_2_, and O_3_
rdpeo	R&D researchers	Person	The number of R&D researchers in a prefecture-level city
Funds	Current assets	Ten thousand yuan	The total amount of current assets of all the enterprises in a prefecture-level city
College	Universities and colleges	Numerical	The number of universities and colleges in a prefecture-level city
Teacher	Teachers	Person	The number of full-time teachers in ordinary colleges and universities
gov	Government expenditure	Ten thousand yuan	The amount of government expenditure on scientific research and technology in a prefecture-level city
FDI	Foreign direct investment	Ten thousand yuan	The amount of foreign capital actually used in the current year
Industry	Industry competition situation	Numerical	The number of industrial enterprises above designated size, which represents market monopoly situation of prefecture-level cities
gdp	Gross Domestic Product	Ten thousand yuan	The gross domestic product of a prefecture-level city
PM_2.5_	PM_2.5_	Metric tons per capita	Content of PM_2.5_ emissions in the air
PM_10_	PM_10_	Metric tons per capita	Content of PM_10_ emissions in the air
Salary	Salary	Yuan	The urban average wage in a prefecture-level city
Student	Students	Person	The number of students in colleges and universities
Patentapply	Patents applied	Piece	The number of patents applied in a prefecture-level city
Invention	Patents for invention	Piece	The number of patents for invention in a prefecture-level city
Patentother	Patents for utility model and industrial design	Piece	The number of patents utility model and industrial design in a prefecture-level city

#### Air quality index

Air quality index (AQI) is calculated from six air pollutants with unified evaluation standards, including PM_2.5_, PM_10_, SO_2_, CO, NO_2_, and O_3_ (92). In this paper, AQI refers to the annual AQI of prefecture-level cities, which obtained from the National Air Quality Daily. The larger the AQI, the more severe the air pollution and the worse the air quality.

#### Regional innovation

This paper uses the number of patents granted (patent) of each prefecture-level city to measure the innovation level. As an intangible asset, patent has great commercial value and is an important way to enhance the competitiveness of enterprises. The quality and quantity of patents are the embodiment of an enterprise's innovation ability and core competitiveness and even the symbol of an enterprise's identity and status in the industry. Therefore, the number of patents granted of a region represents the innovation ability of the region. The larger the number of patents granted, the higher the regional innovation level of prefecture-level cities.

#### Mediators

This paper puts forward two transmission paths, namely, “human capital loss effect” and “funds crowding-out effect.” Therefore, this paper chose the number of R&D researchers (rdpeo) and the scale of current assets (funds) to test the two transmission paths. As the main driving force behind innovation activities, R&D researchers are largely responsible for overall improvements in regional innovation capability. Current assets represent the available funds of a company or a region, which can reflect the change when environmental costs happen.

#### Control variables

This study selects the representative variables of external support, talent support, and economic level of innovation as the control variables (93, 94). The external support of innovation usually refers to the financial support and incentive of non-innovative subjects to innovative enterprises or innovative personnel. The talent support of innovation is the investment and support of the innovation subject, which is mainly composed of university researchers and R&D researchers within enterprises. The economic level is the essential basis to determine whether a region can successfully transform innovation practice into innovation outcomes.

The government expenditure on scientific research and technology (gov) (95, 96) is selected as the government's external support for regional innovation. In reality, the government always directly and indirectly guides the innovation subjects to carry out scientific and technological innovation activities, mainly through financial support method. Therefore, the government's support is measured by the government expenditure on scientific research and technology. As foreign direct investment can bring knowledge spillover and R&D investment, which can affect innovation capability, the amount of foreign capital actually used in the current year (FDI) (97) is employed as the characterization index of regional external investment. The number of colleges and universities (college) (98) and university researchers (teacher) (99) are selected to measure the talent support required for regional innovation. Select gross domestic product of the prefecture-level city (GDP) and number of industrial enterprises above designated size (industry) to represent the economic level and market monopoly of prefecture-level cities (8).

### Model

From the perspective of labor input factors, innovation activities mainly depend on the labor talents with advanced skills and qualities. As the main driving force behind innovation activities, this labor pool is largely responsible for overall improvements in regional innovation capability. Thus, this paper uses the talent supply to reveal this relationship. Additionally, the government support and FDI are the outside force that affects a city's innovation capability. The market environment is the common and outside aspect influencing innovation, which primarily refers to the macroeconomic development level.

To study the effect of air pollution on regional innovation, this paper uses two models, namely, fixed-effect model and mediation model. Fixed-effect model is a panel data analysis method that varies with individuals, but not with time in spatial panel data (3, 4). Fixed-effect model can compare the differences between specific categories or categories of each independent variable and the interaction effects between them and other independent variables. In addition, mediator is an important statistical concept (8). The purpose of studying mediator is to explore the internal mechanism that generates this relationship based on the known relationship between independent and dependent variables (89). Mediation effect analysis is widely used in social science research, such as psychology, management, and communication (56).

In order to test the hypothesis, this paper passed the Hausman test, with the test result *p*-value 0. Therefore, this paper used panel data fixed-effect model for regression to unravel the impact of air pollution on regional innovation. Besides, mediation model is regarded as an effective method to analyze direct and indirect effects and well examine the influencing mechanism of independent variables on dependent variables. This paper set the mediation model according to the model of Zhou et al. (100). The two models are as follows:


(1) $$\begin{array}{l} inno_{it}=\beta_{0}+\beta_{1}AQI_{it}+\beta_{2}gov_{it}+\beta_{3}FDI_{it}+\beta_{4}college_{it}\\ \;+\beta_{5}teacher_{it}+\beta_{6}industry_{it}+\beta_{7}gdp_{it}+u_{i}+\varepsilon_{it} \end{array}$$

(2) $$\begin{array}{l} rdpeo_{it}=\alpha_{0}+\alpha_{1}AQI_{it}+\alpha_{2}gov_{it}+\alpha_{3}FDI_{it}+\alpha_{4}college_{it}\\ \;+\alpha_{5}teacher_{it}+\alpha_{6}industry_{it}+\alpha_{7}gdp_{it}+u_{i}+\varepsilon_{it} \end{array}$$

(3) $$\begin{array}{l} funds_{it}=\gamma_{0}+\gamma_{1}AQI_{it}+\gamma_{2}gov_{it}+\gamma_{3}FDI_{it}+\gamma_{4}college_{it}\\ \;+\gamma_{5}teacher_{it}+\gamma_{6}industry_{it}+\gamma_{7}gdp_{it}+u_{i}+\varepsilon_{it} \end{array}$$

(4) $$\begin{array}{l} inno_{it}=\delta_{0}+\delta_{1}AQI_{it}+\delta_{2}rdpeo_{it}+\delta_{3}gov_{it}+\delta_{4}FDI_{it}\\ \;+\delta_{5}college_{it}+\delta_{6}teacher_{it}+\delta_{7}industry_{it}+\delta_{8}gdp_{it}\\ \;+u_{i}+\varepsilon_{it} \end{array}$$

(5) $$\begin{array}{l} inno_{it}=\eta_{0}+\eta_{1}AQI_{it}+\eta_{2}funds_{it}+\eta_{3}gov_{it}+\eta_{4}FDI_{it}\\ \;+\eta_{5}college_{it}+\eta_{6}teacher_{it}+\eta_{7}industry_{it}+\eta_{8}gdp_{it}\\ \;+u_{i}+\varepsilon_{it} \end{array}$$

where model 1 is the fixed-effect model of air pollution on regional innovation, which is used to test H1. To test H2, models 2 and 4 are derived as the mediation model of human capital loss effect, where model 2 is the effect of air pollution on R&D researchers and model 4 is the effect of R&D researchers loss on regional innovation. Models 3 and 5 are the mediation model of funds crowding-out effect for testing H3, including model 3 for the effect of air pollution on funds and model 5 for the effect of funds on regional innovation. *inno*_*it*_ is the explained variable, which is the logarithmic value of the number of patents granted of city *i* in year *t*; in this paper, *patent*_*it*_ is the proxy variable of *inno*_*it*_; *AQI*_*it*_ is the logarithmic value of the average annual air quality index of a city; *rdpeo*_*it*_ is the number of R&D researchers; *funds*_*it*_ is the amount of current assets; *gov*_*it*_ is the logarithmic value of government expenditure on scientific research and technology; *FDI*_*it*_ is the logarithmic value of the amount of foreign capital actually used in the current year; *college*_*it*_ is the logarithmic value of the number of urban colleges and universities; *teacher*_*it*_ is the logarithmic value of the number of university teachers; *industry*_*it*_ is the logarithmic value of the number of industrial enterprises above designated size; *gdp*_*it*_ is the logarithmic value of the regional gross domestic product; α, β, γ, δ, *and η* are the coefficients of models; *u*_*i*_ is the fixed effect; and ε_*it*_ is the random error. The specific definitions of index and notations are given in Table 2.

## Results

### Descriptive statistics

The descriptive statistics of variables are given in Table 3. Although we all use the logarithmic value of these variables, it is easy to understand the economic implications by using the raw data. Therefore, all the variables in Table 3 are not took logarithm. The average value of patent is 3,438.09, and the variance is 10,697.56, with the maximum value as large as 166,609. It shows that the innovation level of different prefecture-level cities varies greatly, with strong city heterogeneity. The maximum value 166,609 appears in Shenzhen, Guangdong Province. The possible reason is that although Shenzhen is only a prefecture-level city, due to the designation of Shenzhen special zone and the rapid economic development, many high-tech enterprises have settled in Shenzhen, with innovation strongly supported by the government and local enterprises. The maximum value of AQI 230 appears in Leshan, Sichuan Province. All the top ten cities of AQI appear in industrial cities like Leshan. The industry is developed, and the frequent emission of industrial waste gas leads to poor air quality. The minimum value of AQI 18 appears in Wuhai, Inner Mongolia, which is remote and sparsely populated, without large-scale industrial production, and thus has good air quality. There is great heterogeneity for air pollution in different cities. As the external support of regional innovation, the variance of gov and FDI is large, and the regional heterogeneity obviously exists. Among them, the highest gov still appears in Shenzhen, and the highest FDI is Fuzhou, Fujian Province, in addition to Shenzhen, indicating that the two cities have more sufficient external support for innovation. The cities with the lowest gov and FDI appear in Gansu and Tibet, which may be because Gansu and Tibet are remote, economically underdeveloped, and lack of government funds and foreign investment. The average of college is 8.46, while the city with the largest number, Wuhan, has rich educational resources owning 84 colleges and universities, which provides strong support for scientific research. The average proportion of the secondary and tertiary industries is similar, but the focus of developing industries is different; thus, the proportion of the secondary and tertiary industries also changes greatly.

**Table 3:** Descriptive statistics.

**Variable**	**Mean**	**Std. dev**.	**Min**	**Max**
Patent	3,438	10,698	0	166,609
AQI	81	23	18	230
rdpeo	17,776	28,489	14	281,369
Funds	16,400,000	24,400,000	250,786	286,000,000
College	8	14	0	84
Teacher	5,422	9,807	60	67,284
gov	107,830	306,705	753	5,549,817
FDI	84,476	153,986	3	1,400,453
Industry	1,286	1,469	15	11,042
gdp	26,500,000	30,000,000	1,534,089	269,000,000

### Regression results

Table 4 reports the fixed-effect regression results of air quality on regional innovation, which is the results of model 1. Column 1 is the regression result of no control variables, and column 2 reports the regression result of adding the control variables of talent support, external support, and economic level. As can be seen, with the adding of control variables, R-squared has substantial improvement. Column 3 is the regression result of the AQI with one-period lag, which is used to alleviate endogeneity due to the time lag effect of innovation output. Because the air quality of the previous period is not affected by the innovation of the current period, two-way causality is avoided. The results show that the coefficient of AQI is −1.636 at 1% significant level, which represents a 1% increase in AQI may lead to a 1.636% decrease in patent. It indicates that air pollution is significantly detrimental to regional innovation. The coefficient of AQI with one-period lag in column 3 is−1.86, which indicates that air pollution in the past year also has an obvious inhibitory effect on the level of regional innovation. The main reason seems to be that air pollution inhibits regional innovation by dampening the innovation enthusiasm of labor force and raising the production cost of enterprises, which verifies the hypothesis H1. The regression results are also consistent with previous study (101). The impact of air pollution on regional innovation can be described by a simple example. Anshan City in Liaoning Province has poor air quality (AQI > 100). The perennial severe air pollution has greatly affected the health of residents; therefore, its regional innovation level is also affected (patent < 8). But the air quality of Dalian in the same province is good all year round (AQI < 60), which also promotes the generation of innovation in Dalian, with a high level of regional innovation (patent > 10). In addition, government expenditure on scientific research and technology and foreign direct investment can promote the level of regional innovation, mainly by increasing the input of innovation resources from local direct innovation subjects, attracting the inflow of innovation resources from neighboring areas, and increasing the innovation support from local indirect innovation subjects (43). As can be seen from column 2, teacher has a significant positive effect on regional innovation, as university teachers are the main talent reserve for innovation and contribute a lot to patents output. The coefficient of gov is 1.263, which indicates that the innovation is inseparable with the government expenditure on scientific research and technology. Besides, the coefficient of gdp is as large as 19.92, and the main reason for that is gdp is the basic of innovation, which means the funds available for the whole region. But the monopoly has a significant negative effect on regional innovation, because monopoly reduces the motivation and enthusiasm of R&D, which is harmful to innovation.

**Table 4:** Regression results.

**Variables**	**Inno**		
	**(1)**	**(2)**	**(3)**
AQI	−8.871***	−1.636***	
	(0.911)	(0.551)	
L.AQI			−1.860***
			(0.598)
College		0.723	1.316
		(0.838)	(0.931)
Teacher		1.280**	1.472*
		(0.620)	(0.864)
FDI		−0.389***	−0.342**
		(0.118)	(0.134)
gov		1.263***	1.454***
		(0.259)	(0.356)
Industry		−8.356***	−8.419***
		(0.686)	(0.839)
gdp		19.920***	20.690***
		(0.817)	(0.960)
Constant	42.540***	−103.700***	−113.100***
	(3.977)	(7.832)	(9.795)
City and year	Control	Control	Control
R-squared	0.119	0.613	0.572
Number of cities	254	254	254

Robust standard errors in parentheses,

*** p < 0.01,

** p < 0.05,

* p < 0.1.

### Robustness test

To explore whether the negative impact of air pollution still holds, the following robustness tests are carried out, given in Table 5.

**Table 5:** Robustness test.

**Variable**	**(1)**	**(2)**	**(3)**	**(4)**	**(5)**
	**Patent apply**	**patent**	**patent**	**patent**	**patent**
AQI	−0.768***			−1.086***	−2.716***
	(0.152)			(0.306)	(0.879)
PM_2.5_		−0.050***			
		(0.008)			
PM_10_			−0.033***		
			(0.005)		
College	−0.373**	0.683	0.796		1.699*
	(0.188)	(0.830)	(0.847)		(1.014)
Teacher	0.225	0.980	0.924	0.172	2.352***
	(0.163)	(0.597)	(0.593)	(0.215)	(0.895)
FDI	−0.0549***	−0.352***	−0.346***	−0.0613	−0.346**
	(0.018)	(0.112)	(0.110)	(0.053)	(0.145)
gov	0.156***	1.156***	1.139***	0.091	2.899***
	(0.050)	(0.255)	(0.253)	(0.093)	(0.427)
Industry	−0.221**	−7.757***	−7.441***	−0.160	−4.667***
	(0.087)	(0.677)	(0.660)	(0.183)	(0.716)
gdp	0.343***	18.460***	18.190***	1.159***	
	(0.116)	(0.860)	(0.856)	(0.213)	
Salary				0.258	
				(0.169)	
Student					8.949***
					(2.037)
Constant	8.435***	−98.350***	−97.720***	0.166	−132.400***
	(1.351)	(6.896)	(6.794)	(3.361)	(26.540)
City and year	control	control	control	control	control
R-squared	0.196	0.628	0.628	0.146	0.465
Number of cities	254	254	254	254	254

Robust standard errors in parentheses,

*** p < 0.01,

** p < 0.05,

* p < 0.1.

First, holding other variables unchanged, the explained variables are replaced for robustness test, and the number of patents applied (patentapply) is used as the proxy variable of regional innovation level. Because both the number of patents granted and patents applied represent the level of regional innovation, this paper tests the robustness from the perspective of patents applied, and takes patentapply as the dependent variable to test the main results, as shown in column 1 of Table 5. The results showed that there is a significant negative correlation between AQI and patentapply, which implies that air pollution significantly inhibits regional innovation, consistent with the previous result of this paper.

Second, holding other variables unchanged, replace the main explanatory variables and use the annual average PM_2.5_ and PM_10_ emissions as proxy variables of air quality. The regression results are shown in columns 2 and 3. PM_2.5_ and PM_10_, as inhalable particles, are the measurement indicators of air pollution, reflecting the degree of air pollution. The higher the values of PM_2.5_ and PM_10_, the more severe the air pollution. Therefore, this paper takes PM_2.5_ and PM_10_ emissions as independent variables to test the main results. The results show that air pollution (PM_2.5_ and PM_10_) is significantly negatively correlated with inno, which is consistent with the research conclusion of this paper. PM_2.5_ and PM_10_ significantly inhibit regional innovation. The reason may be that the main pollutants affecting the physical condition and innovative thinking of researchers are inhalable particulates, which is harmful to human body.

Third, holding other variables unchanged, replace the control variable for robustness test and replace the number of colleges and universities (college) with the number of students in colleges and universities (student). As the number of students can reflect the potential internal support for innovation, student is used to replace college to show the impact of talent and scientific research reserves on innovation. The results are shown in column 4. The study found that there was a significant negative correlation between AQI and inno, which indicates that air pollution significantly inhibits regional innovation, consistent with the previous result of this paper.

Moreover, the robustness test is carried out by replacing another control variable, namely that the GDP is replaced by the urban average wage (salary). As the urban average wage can better reflect the economic situation of the city and the income level of residents to a certain extent, GDP is replaced for regression. The results are shown in column 5. The study found that there was a significant negative correlation between AQI and inno, which indicates that air pollution significantly inhibits regional innovation, consistent with the main results.

### Heterogeneity analysis

In heterogeneity analysis, we came to the regression results based on model 1 with different groups.

#### Regional development heterogeneity

The above research results imply that air quality has a significant negative impact on regional innovation. Due to the great heterogeneity of air quality and regional innovation level in prefecture-level cities, this paper further divides regions for regression and analyzes the heterogeneity of the impact of air quality on the regional innovation level. Each prefecture-level city is divided into eastern China, central China, and western China according to the development level.

As can be seen from Table 6, columns 1–3 are namely the regression results of eastern China, central China, and western China. Among them, air pollution has a significant inhibitory effect on regional innovation in eastern and central China. The coefficient of AQI in eastern China is −3.650, which means a 1% increase in AQI leads to a 3.650% decrease in patent. The possible reason is the poor conditions of air quality in the eastern and central regions. Air pollution may lead to more strict environmental supervision, and bring a great burden to R&D funds, finally causing the reduction of regional innovation. The inhibitory effect of air pollution on western China is not significant, which may be caused by good air quality in the western remote areas. Even if the air quality becomes worse, it is still within the acceptable range of the human body and has almost no serious impact on human health. Small changes in air quality will not have a substantive impact on regional innovation. Besides, there are few large size cities in western China, which we will discuss later. Therefore, the relationship between innovation and air quality in western China is not significant.

**Table 6:** Regional heterogeneity.

**Variable**	**(1)**	**(2)**	**(3)**
	**Eastern**	**Central**	**Western**
AQI	−3.650*	−1.918**	−0.642
	(1.899)	(0.746)	(0.926)
College	1.628	−1.010	0.864
	(1.158)	(0.884)	(2.269)
Teacher	2.249*	1.088	−0.288
	(1.236)	(0.811)	(1.217)
FDI	0.073	−0.703***	−0.292*
	(0.275)	(0.170)	(0.150)
gov	0.537	2.074***	0.773
	(0.393)	(0.327)	(0.632)
Industry	−9.788***	−6.123***	−5.187*
	(1.166)	(1.021)	(2.616)
gdp	22.640***	16.590***	20.060***
	(1.474)	(1.083)	(1.747)
Constant	−116.000***	−90.930***	−110.600***
	(17.920)	(9.949)	(19.400)
City and year	Control	Control	Control
R-squared	0.664	0.583	0.641
Number of cities	97	110	47

Robust standard errors in parentheses,

*** p < 0.01,

** p < 0.05,

* p < 0.1.

#### City size heterogeneity

The above research results imply that air quality has a significant negative impact on regional innovation. Due to the great heterogeneity of air quality and regional innovation level in prefecture-level cities, this paper further divides regions for regression and analyzes the heterogeneity of the impact of air quality on the regional innovation level. Each prefecture-level city is divided into super city, large city, medium city, and small city according to the size of non-agricultural population in the city.

As can be seen from Table 7, columns 1–4 are namely the regression results of super cities, large cities, medium cities, and small cities. Among them, air pollution has a significant inhibitory effect on regional innovation in super cities, large cities, and medium cities. The possible reason is that the larger the city, the more the concern about the air quality and living environment. Air pollution may bring a great burden to human health, and the deterioration of air quality is as large as 7.478 in super cities at the significant level of 1%, which means a 1% increase in AQI leads to a 7.478% decrease in patent. It indicates that air pollution in super cities is more detrimental to innovation than smaller cities, which makes the environment protect more important in super cities. The inhibitory effect of air pollution in small cities is not significant, which may be caused by the lack of innovation in small cities. Because the size of the cities is small, there are few high-tech researchers or companies; the innovation activity is rare. Therefore, the relationship between innovation and air quality in small cities is not significant. It provides evidence for the previous result of western China.

**Table 7:** Size heterogeneity.

**Variable**	**(1)**	**(2)**	**(3)**	**(4)**
	**Super**	**Large**	**Medium**	**Small**
AQI	−7.478**	−4.530**	−5.384***	−0.244
	(2.805)	(1.852)	(1.819)	(0.697)
College	−1.973	0.377	0.179	1.015
	(3.006)	(3.638)	(1.376)	(1.430)
Teacher	2.914	1.969	1.774*	0.245
	(5.384)	(1.691)	(1.011)	(0.891)
FDI	−0.394	−0.293	−0.500**	−0.164
	(0.483)	(0.240)	(0.210)	(0.161)
gov	1.503*	1.599**	0.748**	0.758*
	(0.774)	(0.655)	(0.353)	(0.386)
Industry	−11.630***	−9.441***	−7.514***	−5.634***
	(1.085)	(1.236)	(1.743)	(1.396)
gdp	20.760***	20.050***	25.040***	18.630***
	(1.993)	(2.238)	(1.653)	(1.101)
Constant	−90.690*	−96.730***	−123.200***	−101.200***
	(51.020)	(21.030)	(17.920)	(12.930)
City and year	Control	Control	Control	Control
R-squared	0.690	0.620	0.612	0.634
Number of cities	30	46	109	69

Robust standard errors in parentheses,

*** p < 0.01,

** p < 0.05,

* p < 0.1.

#### Air quality heterogeneity

The above research results imply that air quality has a significant negative impact on regional innovation. However, whether the air pollution affects regional innovation differently due to the air quality conditions in cities deserves our discussion. This paper yearly divides cities to cities with good air quality, cities with general air quality and cities with poor air quality according to the air quality condition.

As can be seen from Table 8, columns 1–3 are namely the regression results of cities with good, general, and poor air quality. The result shows that the air quality has a significant negative effect on regional innovation in cities with poor air quality, even in cities with general air quality. The coefficient of AQI −5.204 with the significant level of 1% shows the strong detrimental effect of air quality on innovation, which deserves the attention of relevant departments. A 1% increase in AQI will lead to a 5.204% decrease in regional innovation. The coefficient of AQI is predictably insignificant in cities with good air quality due to the good foundation of air quality in the cities. Even if the air quality becomes worse, it is still within the acceptable range of the human body and has almost no serious impact on human health. Small changes in air quality will not have a substantive impact on regional innovation. Therefore, the effect is insignificant in cities with good air quality.

**Table 8:** Air quality heterogeneity.

**Variable**	**(1)**	**(2)**	**(3)**
	**Good**	**General**	**Poor**
AQI	2.844	−5.707***	−5.204***
	(1.898)	(2.129)	(1.591)
College	−0.105	3.266**	−0.449
	(1.096)	(1.262)	(1.571)
Teacher	3.440***	0.561	1.083
	(1.205)	(1.276)	(1.500)
FDI	−0.405***	−0.159	−0.333
	(0.139)	(0.370)	(0.269)
gov	1.230***	1.553**	1.109**
	(0.396)	(0.613)	(0.477)
Industry	−2.437*	−9.558***	−7.948***
	(1.343)	(1.182)	(1.205)
gdp	17.890***	20.190***	20.380***
	(1.610)	(1.693)	(1.681)
Constant	−156.700***	−84.290***	−90.630***
	(14.410)	(18.020)	(20.340)
City and year	Control	Control	Control
R-squared	0.575	0.581	0.654
Number of cities	147	167	164

Robust standard errors in parentheses,

*** p < 0.01,

** p < 0.05,

* p < 0.1.

### Transmission path analysis

The above research implies that air quality has a significant detrimental impact on regional innovation. According to the above analysis, there are two transmission paths for the negative effect, namely, “human capital loss effect” and “funds crowding-out effect.”

#### Human capital loss effect

Air pollution may lead to dampening the enthusiasm to innovation of labor force and the loss of labor force. In this section, this paper uses the mediation model to test human capital loss effect through models 2 and 4. The regression result is given in Table 9. In column 2, the coefficient of AQI is −0.0854 and significant statistically, indicating that air pollution is negatively and significantly correlated with the innovative researchers. A 1% increase in AQI leads to a 0.0854% decrease in patent. The results in column 3 show that the coefficient of AQI is negative and significant statistically at the level of 1%, and the coefficient of rdpeo is positive and significant statistically at the level of 1%, thereby supporting hypothesis H2. Air pollution leads to the loss of R&D researchers and then causes the reduction of innovation output, reflected in the number of patents granted.

**Table 9:** Human capital loss effect.

**Variable**	**(1)**	**(2)**	**(3)**
	**Patent**	**rdpeo**	**Patent**
AQI	−1.636***	−0.085*	−0.556***
	(0.551)	(0.044)	(0.112)
rdpeo			0.267***
			(0.038)
College	0.723	0.471***	0.225**
	(0.838)	(0.040)	(0.089)
Teacher	1.280**	−0.007	0.016
	(0.620)	(0.045)	(0.067)
FDI	−0.389***	−0.009**	−0.046**
	(0.118)	(0.005)	(0.019)
gov	1.263***	0.016	0.241***
	(0.259)	(0.016)	(0.038)
Industry	−8.356***	−0.054	0.640***
	(0.686)	(0.035)	(0.059)
gdp	19.920***	0.200***	0.027
	(0.817)	(0.033)	(0.093)
Constant	−103.700***	6.122***	0.596
	(7.832)	(0.380)	(0.599)
City and year	Control	Control	Control
R-squared	0.613	0.275	0.821
Number of cities	254	254	254

Robust standard errors in parentheses,

*** p < 0.01,

** p < 0.05,

* p < 0.1.

#### Funds crowding-out effect

The severity of regional air pollution is likely to make the local government increase the environmental requirements, which reduces the available funds of enterprises and then forces enterprises to reduce investment in R&D and innovation. In this section, this paper uses the mediation model to test funds crowding-out effect through models 3 and 5. The regression result is given in Table 10. In column 2, the coefficient of AQI is −0.0599 and significant statistically, indicating that air pollution is negatively and significantly correlated with the innovation funds. The results in column 3 show that the coefficient of AQI is negative and significant statistically at the level of 1%, and the coefficient of rdpeo is positive and significant statistically at the level of 1%, thereby supporting hypothesis H3. Air pollution may increase the environmental cost and salary required, thereby increase the operating costs, and reduce the available funds. The reduction in the available funds eventually leads to the reduction in patents output.

**Table 10:** Funds crowding-out effect.

	**(1)**	**(2)**	**(3)**
**Variable**	**Patent**	**Funds**	**Patent**
AQI	−1.636***	−0.060*	−1.460***
	(0.551)	(0.033)	(0.520)
Funds			2.829***
			(0.723)
College	0.723	−0.058	0.911
	(0.838)	(0.042)	(0.840)
Teacher	1.280**	0.043	1.131*
	(0.620)	(0.040)	(0.592)
FDI	−0.389***	−0.012	−0.355***
	(0.118)	(0.009)	(0.116)
gov	1.263***	0.036**	1.153***
	(0.259)	(0.016)	(0.248)
Industry	−8.356***	0.112**	−8.670***
	(0.686)	(0.055)	(0.653)
gdp	19.920***	0.518***	18.440***
	(0.817)	(0.054)	(0.930)
Constant	−103.700***	11.170***	−134.900***
	(7.832)	(0.515)	(10.480)
Observations	1,489	1,486	1,486
R-squared	0.613	0.249	0.624
Number of cities	254	254	254

Robust standard errors in parentheses,

*** p < 0.01,

** p < 0.05,

* p < 0.1.

## Discussion

This paper reported the effect of air pollution on regional innovation among different regions and economic development levels. Consistent with previous studies (19, 28), this paper investigated the negative effect of air pollution on regional innovation and found that air pollution has more significant detrimental impact on the innovation level for those heavily polluted regions. Due to the high cost and periodicity of technological innovation (102), as well as strong path dependence, a large amount of production capacity in cities with serious air pollution is locked in high pollution, high emission, and low value-added industries. However, different from Lin et al. (8), this paper studied the human capital loss effect with the significant coefficient of AQI −0.0854 and found that air pollution negatively affects regional innovation through the human capital loss effect at the prefecture level, not at the provincial level. Besides, this paper investigated the regions which will suffer from the more severe and significant impact of air pollution on regional innovation, indicating that air pollution in developed regions and larger-scale regions is more detrimental to the innovation level. The effects of developed regions and larger-scale regions are interrelated, with the mutually corroborated empirical results, since the more developed regions, the larger scale of population, and city size.

Although valuable, this study still has some limitations. First, it only estimated the model using the panel data for the period 2014–2019. Future studies could be based on longer datasets, 20 years for example. Second, this study only examined the impact of air pollution on patents granted. Although patents granted are the typical representative and straightforward indicator for the regional innovation level, the study still needs to be extended to more flexible and comprehensive index in order to obtain greater generality of the results and further insights. Moreover, introducing more potentially relevant factors, improving the mediation model, and exploring more influencing paths could be valuable research activities.

To minimize the negative impacts of air pollution on regional innovation, it is necessary to revisit the hypotheses tested and recognize the role of the hypotheses to the air pollution. This section further discusses several related factors that may influence the impact of air pollution on regional innovation and determine how can reduce the negative impacts.

### Technology

According to catch-up theory, the standard catch-up cycle for which developing regions to gain technology from developed regions consists of four stages, namely, entry, gradual catching-up, forging ahead, and falling behind (52). Long innovation cycle and large technology gap limit the likelihood of the occurrence of the catch-up cycles and the development of innovation in developing regions. The pollution haven hypothesis argues that the removal of trade barriers leads to the relocation of environmental harmful production staging from high-income countries with stringent environmental regulation to developing countries with less stringent environmental regulation. To catch up with developed regions, developing regions have to bear the aggravation of air pollution. As a result, the initial time of forming clean technology in developing regions is delayed, and it will aggravate the negative effect of air pollution on regional innovation. In this context, the long-life cycle of clean technology innovation means that the technology ages slowly. This suggests that it is difficult for latecomers like developing regions to acquire outdated technology to utilize for capability building, which leads to the decrease in the innovation level in developing regions.

### Land-use changes

With the air pollution in China getting worse, land-use change becomes one of the key factors that affect air pollution. In recent years, land cover has changed rapidly in developing nations, particularly in China (103). The rapid changes in land cover are often characterized by urban sprawl, farmland displacement, and deforestation, leading to the loss of arable land, habitat destruction, and the decline of the natural greenery areas (104). With the development of urbanization, the consumption of resources and energy has increased, the level of biodiversity has decreased, environmental pollution is approaching the critical level, and the contradiction between human habitat activity and ecological environment has become increasingly prominent (105). These losses have a substantial impact on urban environmental conditions and aggravate air pollution in these regions. Many factors could cause air pollution, such as construction dust, domestic garbage, and vehicle exhaust, but most air pollution can be reflected by land-use changes. There are mainly six land-use types, namely, water, woodland, grassland, cultivated land, urban, and unused land. In recent years, the dominant factor affecting air quality in land use changes transitions from ocean, to woodland, to urban land, and eventually into grassland or unused land when moving from the coast to inland China. Due to rapid economic development, developed regions like eastern China have shown faster urban sprawl and large vegetation areas have been replaced by city buildings over the last 10 years, leading to an increase in air pollution, which aggravates the detrimental effect of air pollution on regional innovation.

### Innovation types

This paper uses patents granted as the proxy variable of regional innovation. There are two types of innovation, namely, radical innovation and incremental innovation. A patent is a type of long-term literature containing the most complete design information in most fields. Thus, it can provide designers with valuable guides for solving various design problems in different fields. There are three types of patents, namely, patents for invention, patents for utility model, and industrial design patents. A patent for invention refers to a new technical proposal for a product, method, or improvement thereof. The patent for utility model refers to a new technical solution suitable for practical use proposed for the shape, structure, or combination of products. Industrial design patent refers to a new design of the shape, pattern, or combination thereof and the combination of color, shape, and pattern, which is esthetic and suitable for industrial application. Therefore, it is obvious that patents for invention are the most important type of patents, which represents novelty, creativity, and practicality. As the exploration of unknown paths, invention generally has the characteristics of long cycle, great uncertainty, high failure rate, but huge potential benefits, which is a breakthrough innovation. Therefore, in order to test the impact of innovation types on the detrimental effect of air pollution, this paper further divided patents into two types: one is patents for invention and the other is patents for utility model and industrial design.

Table 11 reports the regression results of the two innovation types. Columns 1–2 are the results of radical innovation and incremental innovation, respectively. As can be seen from column 1, the coefficient of AQI is −0.698 at the significant level of 10%, which indicates a 1% increase in air pollution will lead to a 0.698% decrease in inventions. Column 2 indicates that a 1% increase in air pollution will lead to a 1.475% decrease in patents for utility model and industrial design. The coefficients of air pollution on both types of innovation are significant, arguing that air pollution is detrimental to all types of regional innovation. For instance, as Tangshan city is an industrial city in Hebei Province, the total number of patents in 2017 is 3,677, with the number of inventions 640. As Tangshan is an industrial city, the patents mainly relate to manufacturing, such as the award-winning patent “A control method and device for switch-type electro-pneumatic valve of EMU.” But as Dalian is a coastal city in Liaoning Province, the total number of patents is higher as 7,768, with the number of inventions 2,604. It has more patents and inventions than Tangshan, and the patents mainly relate to new energy and power, such as the award-winning patent “Power integration system arranged under the vehicle of High-speed Diesel Multiple Unit.”

**Table 11:** Innovation types.

**Variable**	**(1)**	**(2)**
	**Invention**	**Patentother**
AQI	−0.698*	−1.475***
	(0.369)	(0.181)
College	−0.333	−0.466
	(0.562)	(0.283)
Teacher	−0.061	0.410*
	(0.302)	(0.216)
FDI	−0.020	−0.019
	(0.024)	(0.018)
gov	−0.072	0.253***
	(0.090)	(0.065)
Industry	−0.112	−0.402***
	(0.167)	(0.111)
gdp	0.188	0.989***
	(0.240)	(0.174)
Constant	9.975***	4.266***
	(3.101)	(1.585)
City and year	Control	Control
Observations	720	720
R-squared	0.032	0.317
Number of cities	252	252

Robust standard errors in parentheses,

*** p < 0.01,

* p < 0.1.

### Governmental policies

According to the funds crowding-out effect, air pollution tends to increase the requirements and punishment mechanism for environmental governance, thereby influencing regional innovation. Government policies are also important factors which influence both air pollution and regional innovation. Therefore, this paper uses the policy China Carbon Emission Trade Exchange (CCETE) in 2017 as a sample to test the effect of government policies. CCETE is one of the core policies to achieve the carbon peak and carbon neutral goals. Since 2011, Beijing, Tianjin, Shanghai, Chongqing, Guangdong, and Hubei have carried out carbon emission trading pilot work. At the end of 2017, China launched the trading of carbon emission rights. In the short run, when the trading of carbon emission rights begins, enterprises in these regions should buy the rights once they need emissions, increasing the production costs and leading to the decrease in R&D expenditure. Thus, this paper uses the difference-in-difference (DID) model to test the policy effect, through taking the prefecture-level cities that have conducted the pilot before 2017 as the control group, and the prefecture-level cities that have not conducted the pilot as the treated group.

Table 12 reports the difference-in-difference (DID) result of the effect of CCETE on regional innovation. The coefficient of the interactive item treat^*^time is −0.876, which means the CCETE policy is significantly detrimental to regional innovation in the short run. The possible reason is the trading of carbon emission rights makes enterprises increase the production costs in the short run, but in the long run, the launch of this policy will encourage the low polluted enterprises and lead to the clean technology innovation. The mechanism of CCETE controls the industrial pollution gross by punishing the heavily polluted firms and guerdon the low polluted firms.

**Table 12:** Government policies effect.

**Variables**	**(1)**
Time	8.391***
	(0.110)
Treat	0.300***
	(0.078)
Treat*time	−0.876***
	(0.118)
College	0.052
	(0.087)
Teacher	0.002
	(0.069)
FDI	0.026
	(0.017)
gov	0.299***
	(0.039)
Industry	0.227***
	(0.049)
gdp	0.075
	(0.085)
Constant	−5.826***
	(0.446)
City and year	control
Observations	1,489
R-squared	0.950

Robust standard errors in parentheses,

*** p < 0.01.

## Conclusion and policy implications

Air pollution not only hinders human health and steady economic growth, but also inhibits the innovation level, which is the key factor to realize sustainable development. Therefore, this paper uses the fixed-effect model and mediation model to analyze the impact of air pollution on regional innovation and the transmission paths through the data of 285 prefecture-level cities in China from 2014 to 2019. Considering the regional heterogeneity, this paper also divides the samples according to development level, city size, and air quality. Besides, patents granted are divided into two innovation types to determine the type of enterprises affected by air pollution and the impact of them. The following conclusions can be drawn. First, air pollution has a significant detrimental effect on regional innovation. The overall regional innovation level of the city decreases by 1.636% when a city's air pollution increases by 1%. With the intensification of air pollution, the level of regional innovation declines significantly. There are also differences between innovation types, and the detrimental effect is more severe on incremental innovation compared with radical innovation. Second, air pollution produces significantly negative impacts on innovation funds and researchers and further produces significantly indirect negative impacts on regional innovation. It means air pollution negatively affects regional innovation through the human capital loss effect and funds crowding-out effect. Third, as for development level heterogeneity, air pollution has a significant inhibitory effect among eastern and central China, but has not in western China. Fourth, the negative effect of air pollution on regional innovation is significant in large- and medium-sized cities with the coefficient of super cities as large as 7.478, which is 1.5 times of large cities. Besides, air pollution has significant negative impact on regional innovation in cities with poor and general air quality, except cities with good air quality.

Based on the above, this paper has some policy implications. First, governments should develop targeted environmental policies and implement effective measures to control air pollution and improve air quality to attract talents. When formulating and implementing relevant legislation, policymakers in large- and medium-sized developed cities and cities with poor or general air quality condition should be aware of the use of mandatory measures to improve air quality, such as traffic restrictions and shutdown. Policymakers in super cities should specially focus on air pollution governance for the severe impact of air pollution on innovation. For underdeveloped cities with good air quality, policymakers can actively attract talents with good air quality as an advantage, gradually promoting innovation. Second, policymakers should further strengthen the support for innovation activities in various ways, such as innovation grants and talent subsidies, and induce firms to increase capital investment and personnel input in innovation. Third, firms should provide good development conditions and environment for innovative talents and may even give appropriate pollution subsidies, so as to increase loyalty and stickiness of talents. Besides, government could allocate substantial financial resource to research and development for innovating environmental friendly production technologies and renewable energy sources. Policymakers could finally control the industrial pollution gross by trade-off of punishing the heavily polluted firms and guerdon the low polluted firms. Finally, the impact of air pollution on incremental innovation enterprises is particularly serious, which requires the regional air quality should be carefully considered in location by such enterprises.

## Data availability statement

The original contributions presented in the study are included in the article/Supplementary material, further inquiries can be directed to the corresponding authors.

## Author contributions

BC and CT were involved in conceptualization. ZL was involved in methodology, software, data curation, and resources. ZL, MH, and BC were involved in validation. LG and CT were involved in formal analysis. MH was involved in investigation. ZL and CT were involved in writing—original draft preparation. LG, BC, CT, and MH were involved in writing—review and editing. RA and MH were involved in visualization. BC was involved in supervision, project administration, and funding acquisition. All authors have read and agreed to the published version of the manuscript.

## Funding

This research was funded by the National Natural Science Foundation of China (Grant No. 71873016).

## Conflict of interest

The authors declare that the research was conducted in the absence of any commercial or financial relationships that could be construed as a potential conflict of interest.

## Publisher's note

All claims expressed in this article are solely those of the authors and do not necessarily represent those of their affiliated organizations, or those of the publisher, the editors and the reviewers. Any product that may be evaluated in this article, or claim that may be made by its manufacturer, is not guaranteed or endorsed by the publisher.
