# Supplementary material for: Is air pollution detrimental to regional innovation? An empirical heterogeneity test based on Chinese cities
Source: Front Public Health. 2022 Nov 21;10:981306. doi: 10.3389/fpubh.2022.981306 (PMC9720137; doi:10.3389/fpubh.2022.981306)
Supplement: Supplementary file 1 [file Table_1.DOCX]

The Hausmann test result of the fixed effect model is shown below.
